# Pediatric epilepsy surgery: Global survey of invasive explorations

**DOI:** 10.1002/epi.70325

**Published:** 2026-06-11

**Authors:** Georgia Ramantani, Martha Feucht, Dorottya Cserpan, Javier Aparicio, Alexis Arzimanoglou, Fabrice Bartolomei, William Bingaman, Kees P. J. Braun, Richard J. Burman, Dezhi Cao, Roberto Caraballo, Mathilde Chipaux‐Raffo, Thomas Cloppenborg, J. Helen Cross, Arthur Cukiert, Cristine Mella Cukiert, Daniel J. Curry, Luca De Palma, Petia Dimova, Christin Eltze, Dario J. Englot, Johannes M. Nico Enslin, Aria Fallah, Stefano Francione, Deepak Gill, Tove Hallböök, A. Simon Harvey, Hans Holthausen, Sita Jayalakshmi, Yuwu Jiang, Lakshminarayanan Kannan, Matt Lallas, Sandeep Bhagwan Patil, María Ángeles Pérez‐Jiménez, Juan Carlos Pérez‐Poveda, Tom Pieper, Sabine Rona, Victoria San Antonio‐Arce, Jay Shetty, Mary Lou Smith, Piradee Suwanpakdee, Oana Tarta Arsene, Laura Tassi, Martin Tisdall, Manjari Tripathi, Ahsan Moosa Naduvil Valappil, Howard Weiner, Elaine Wirrell, Lixin Cai, Sarah Ferrand‐Sorbets, ILAE Pediatric Epilepsy Surgery Taskforce and Study Group, Carmen Barba, Marcelo Budke, James Butler, Poodipedi Sarat Chandra, Mary Connoly, Sameer Dal, Georg Dorfmüller, Noelle Enright, Siby Gopinath, Aris Hadjinicolaou, Masaki Iwasaki, Thilo Kalbhenn, Kalman A. Katlowitz, Kensuke Kawai, Takayuki Kikuchi, Katarzyna Kotulska‐Jóźwiak, Niklaus Krayenbühl, Martin Kudr, Noritsugu Kunihiro, Cassia Maniquis, Ailsa McLellan, Carolina Munoz Castro, Lino Nobili, Wim Otte, Manas Panigrahi, Veronica Pelliccia, Karl Rössler, Siraruj Sakoolnamarka, Didier Scavarda, Catalina Soto Órdenes, Nicola Specchio, Nigel Peter Symss, TS Dhanya, Olga Teo, Hartlieb Till, Dilşad Türkdoğan, Takehiro Uda, Vrajesh Udani, Shimrit Uliel‐Sibony, Naotaka Usui, Carlos Valera, Lejla Vendegh, Yi Wang, Jithangi Wanigasinghe, Jo Wilmshurst, Oscar Zorro Guio

**Affiliations:** ^1^ Department of Neuropediatrics University Children’s Hospital, University of Zurich Zurich Switzerland; ^2^ Children’s Research Center University Children’s Hospital Zurich Zurich Switzerland; ^3^ Neuroscience Center Zurich Swiss Federal Institute of Technology Zurich and University of Zurich Zurich Switzerland; ^4^ University of Zurich Zurich Switzerland; ^5^ Department of Pediatrics Medical University of Vienna, Member of the European Reference Network for Rare and Complex Epilepsies Vienna Austria; ^6^ Epilepsy Unit Hospital Sant Joan de Déu, Member of the European Reference Network for Rare and Complex Epilepsies Barcelona Spain; ^7^ Epileptology and Cerebral Rhythmology, Assistance Publique–Hôpitaux de Marseille, Hôpital de la Timone, and Institut de Neurosciences Des Systèmes, Inserm Aix Marseille Université Marseille France; ^8^ Epilepsy Center, Neurological Institute Cleveland Clinic Cleveland Ohio USA; ^9^ Department of Child Neurology, University Medical Center Utrecht Brain Center University Medical Center Utrecht Utrecht the Netherlands; ^10^ Division of Neurosurgery, Department of Surgery, Neuroscience Institute University of Cape Town Cape Town South Africa; ^11^ Epilepsy Center Shenzhen Children’s Hospital Shenzhen China; ^12^ Department of Neurology Hospital de Pediatría Prof. Dr. Juan P. Garrahan Buenos Aires Argentina; ^13^ Pediatric Neurosurgery Department, Rothschild Foundation Hospital Centre de Compétence Maladies Rares Epilepsies Rares, Member of the European Reference Network for Rare and Complex Epilepsies Paris France; ^14^ Department of Epileptology, Bethel Epilepsy Center, Medical School Ostwestfalen‐Lippe Bielefeld University Bielefeld Germany; ^15^ Great Ormond Street Hospital for Children National Health Service Trust, Developmental Neurosciences University College London Great Ormond Street Institute of Child Health London UK; ^16^ Clinica Cukiert São Paulo Brazil; ^17^ Division of Pediatric Neurosurgery, Department of Surgery Texas Children’s Hospital Houston Texas USA; ^18^ Department of Neurosurgery Baylor College of Medicine Houston Texas USA; ^19^ Neurology, Epilepsy, and Movement Disorders Unit, Bambino Gesù Children’s Hospital Istituto di Ricovero e Cura a Carattere Scientifico, Member of the European Reference Network for Rare and Complex Epilepsies Rome Italy; ^20^ Functional and Epilepsy Surgery Center University Hospital St. Ivan Rilski Sofia Bulgaria; ^21^ Department of Neurological Surgery Vanderbilt University Medical Center Nashville Tennessee USA; ^22^ Division of Neurosurgery, Department of Surgery, Neuroscience Institute University of Cape Town and Red Cross War Memorial Children’s Hospital Cape Town South Africa; ^23^ Department of Neurosurgery David Geffen School of Medicine at the University of California Los Angeles California USA; ^24^ Child Neuropsychiatry Unit Istituto di Ricovero e Cura a Carattere Scientifico, Istituto Giannina Gaslini Genoa Italy; ^25^ Children’s Hospital at Westmead Sydney New South Wales Australia; ^26^ Queen Silvia Children’s Hospital Sahlgrenska University Hospital Gothenburg Sweden; ^27^ Royal Children’s Hospital Melbourne Victoria Australia; ^28^ Center for Pediatric Neurology, Neurorehabilitation, and Epileptology Schön Clinic Vogtareuth Germany; ^29^ Department of Neurology Krishna Institute of Medical Sciences Secunderabad India; ^30^ Pediatric Epilepsy Center Peking University First Hospital Beijing China; ^31^ Gleneagles Hospital Chennai India; ^32^ Department of Neurology Nicklaus Children’s Hospital Miami Florida USA; ^33^ Deenanath Mangeshkar Hospital and Research Center Pune India; ^34^ Department of Clinical Neurophysiology Niño Jesús University Children’s Hospital Madrid Spain; ^35^ Hospital Universitario San Ignacio and Pontificia Universidad Javeriana Bogotá Colombia; ^36^ Swiss Epilepsy Center Zurich Switzerland; ^37^ Freiburg Epilepsy Center, Medical Center–University of Freiburg, Member of the European Reference Network for Rare and Complex Epilepsies, and Faculty of Medicine University of Freiburg Freiburg Germany; ^38^ Royal Hospital for Children and Young People Edinburgh UK; ^39^ Hospital for Sick Children and University of Toronto Mississauga Toronto Ontario Canada; ^40^ Department of Pediatrics Phramongkutklao Hospital Bangkok Thailand; ^41^ Clinical Hospital of Psychiatry and Pediatric Neurology Unit/Department of Neurosurgery Marie Skłodowska Curie Emergency Hospital for Children Bucharest Romania; ^42^ Claudio Munari Epilepsy Surgery Center Niguarda Hospital Milan Italy; ^43^ Department of Neurology All India Institute of Medical Sciences New Delhi India; ^44^ Division of Child and Adolescent Neurology and Epilepsy, Department of Neurology Mayo Clinic Rochester Minnesota USA; ^45^ Peking University First Hospital Beijing China; ^46^ Rothschild Foundation Paris France

**Keywords:** global survey, invasive EEG, MRI‐negative epilepsy, pediatric epilepsy surgery, SEEG

## Abstract

**Objective:**

Invasive presurgical evaluation plays a key role in pediatric epilepsy surgery, particularly in magnetic resonance imaging (MRI)‐negative cases, by guiding resective, disconnective, or ablative procedures. This International League Against Epilepsy (ILAE) Pediatric Epilepsy Surgery Taskforce study provides an updated global overview of current invasive evaluation practices.

**Methods:**

Group‐level data were collected from 61 epilepsy surgery programs (49 pediatric‐only) in 29 countries across six continents. Included were children and adolescents who underwent presurgical evaluation and epilepsy surgery in 2023. The study was designed to enable comparison with the similar ILAE survey conducted in 2004.

**Results:**

A total of 2427 patients were included. Invasive evaluations were performed in 21.1% of cases, most frequently in North America (33.7%, higher than Europe: 18.0%, *p* = .003). Among invasive cases, 32.3% had no detectable MRI abnormalities. The main indication for invasive evaluation was seizure onset localization (88.1%), followed by motor or sensory mapping (17.2%) and language mapping (14.2%). Stereoelectroencephalography (SEEG) was the predominant technique (19.8% overall, 93.6% of invasive cases), more common in North America (30.0%, *p* = .021) and less common in South America (7.4%, *p* < .001). Subdural electrodes were used in only 3.2% of invasive cases, and combined depth and subdural approaches in 3.2%. SEEG‐guided radiofrequency thermocoagulation (RF‐TC) was performed in 40.9% of SEEG cases, most commonly in Asia (63.8%). In 16.2% of invasive evaluations, patients did not proceed to resection, disconnection, or ablation, with the highest rate in Europe (28.5%).

**Significance:**

This global survey provides the first broad overview of invasive evaluation practices in pediatric epilepsy surgery across participating centers worldwide. It highlights the widespread adoption of SEEG, declining use of subdural electrodes, and increasing application of SEEG‐guided RF‐TC. The high proportion of MRI‐negative cases and the considerable proportion of patients not proceeding to resection, disconnection, or ablation underscore the complexity of contemporary surgical candidates and the need for further refinement of selection strategies.

**Key points**

- Data from 61 epilepsy surgery programs in 29 countries (*n* = 2427) provide an international overview of invasive evaluation practices in pediatric epilepsy surgery.
- Invasive evaluations were performed in 21.1% of cases, with the highest use in North America (33.7%).
- MRI was negative in 32.3% of patients who underwent invasive evaluation.
- SEEG was the predominant modality (93.6%); subdural and combined depth and subdural electrode approaches were rarely used.
- SEEG‐guided radiofrequency thermocoagulation was performed in 40.9% of SEEG cases, most commonly in Asia (63.8%).
- In 16.2% of invasive evaluations, patients did not proceed to resection, disconnection, or ablation, with the highest rate in Europe (28.5%).

## 1 INTRODUCTION

Pediatric epilepsy surgery is an evidence‐based intervention^1^, ^2^ and is increasingly utilized worldwide.^3^, ^4^, ^5^, ^6^, ^7^, ^8^, ^9^ The 2004 International League Against Epilepsy (ILAE) survey provided the first global overview of pediatric epilepsy surgery, with data from 543 patients across 20 centers in North America, Europe, and Australia.^10^ Although that report covered broad aspects of presurgical evaluation and surgical care, it included only limited detail on invasive presurgical evaluation.

Since then, access to epilepsy surgery has expanded globally, not only in high‐income countries^11^ but also in low‐ and middle‐income settings.^6^ The surgical population has also evolved; advances in neuroimaging^12^ and genetic diagnostics^13^ have improved candidate selection,^14^ and more children without magnetic resonance imaging (MRI)‐visible lesions or with extensive abnormalities are now considered for surgery.^15^, ^16^ The widespread adoption of stereoelectroencephalography (SEEG) has further reshaped presurgical strategies, offering a better tolerated and less invasive alternative to subdural electrodes, with lower complication rates and comparable seizure outcomes in pediatric cohorts.^17^, ^18^

Despite these developments, international data on invasive presurgical evaluation in the pediatric population remain limited. Recent reports have described national or regional practices,^17^ but no comprehensive international overview exists.

To address this gap, the ILAE Pediatric Epilepsy Surgery Taskforce within the Surgical Therapies Commission 2021–2025 conducted an international survey of invasive presurgical evaluation in children undergoing epilepsy surgery in 2023. This study provides an updated overview of current practices, regional variation, and recent trends, with the goal of informing clinical practice, guideline development, and future research in pediatric epilepsy surgery.

## 2 MATERIALS AND METHODS

### 2.1 Study design

This international cross‐sectional survey collected group‐level data on children and adolescents (≤18 years) who underwent epilepsy surgery in 2023. Included were patients who underwent either (1) invasive presurgical evaluation or (2) therapeutic procedures such as resection, disconnection, ablation, or neuromodulation. Postsurgical seizure, developmental, and behavioral outcomes were not assessed.

### 2.2 Survey development and scope

A web‐based data entry form was developed and hosted on REDCap servers at the University Children's Hospital Zurich. The form was designed to collect standardized, deidentified group‐level clinical data on pediatric epilepsy surgery cases treated in 2023.

### 2.3 Participating centers and inclusion criteria

To expand global representation compared to the 2004 ILAE survey, epilepsy surgery centers from all continents were invited to participate. Eligible centers included pediatric‐only programs and mixed‐age centers with dedicated pediatric services. Centers were identified through the ILAE Pediatric Epilepsy Surgery Taskforce, the ILAE Surgical Therapies Commission, and regional ILAE networks. Participating centers were required to provide group‐level data on all pediatric patients (≤18 years) who underwent invasive evaluation or epilepsy surgery in 2023. Centers were excluded if their pediatric surgical volume consisted primarily or exclusively of vagal nerve stimulator implantations.

### 2.4 Data collection

Each center appointed one or more local leads responsible for extracting and entering data into REDCap. Collected variables included the use, indication, and modality of invasive evaluations and whether these were followed by resective, disconnective, or ablative surgery.

### 2.5 Statistics

Descriptive statistics were calculated for all study variables. Continuous variables are reported as means and SDs, categorical variables as frequencies and percentages.

Group comparisons across continents were assessed using generalized estimating equations (GEE). For continuous outcomes, a Gaussian family with identity link was applied; for proportional outcomes, a binomial family with logit link was used. Models were fitted using the *geeglm()* function from the geepack package in R. To account for intracountry clustering, centers were grouped by country using an exchangeable correlation structure. Observations were weighted by the number of patients per center. Europe served as the reference category for all continent‐level comparisons. Africa was excluded from inferential analyses due to small sample size (*n* = 17). For outcomes with few events, models could be fitted but estimates were imprecise (wide confidence intervals) and should be interpreted cautiously. Where zero or sparse counts yielded unstable GEE estimates, results are deemed not estimable; these are provided in the Supporting Information Tables (S1–S3) but are not discussed in the main text.

Statistical significance was defined as a two‐sided *p*‐value ≤ .05. All analyses were performed using R (v. 4.3.1).^19^

## 3 RESULTS

### 3.1 Participating centers and patient cohort

**FIGURE 1:**
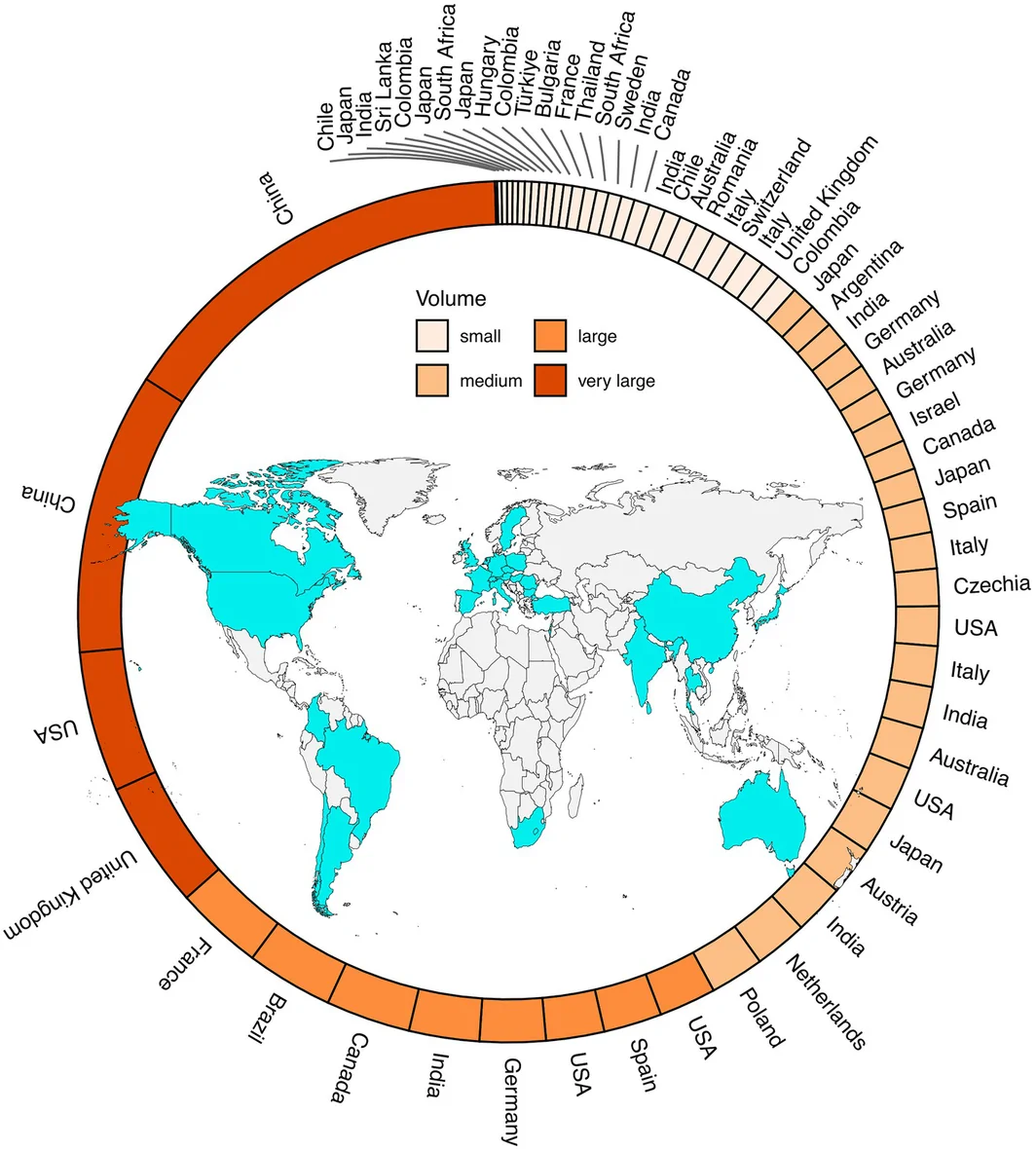
Global distribution of contributing epilepsy surgery programs. Data were contributed by 29 countries across six continents (highlighted in cyan), representing a total of 2427 pediatric patients who underwent epilepsy surgery in 2023. The central map illustrates the geographic spread of participating countries. The surrounding ring is subdivided into segments corresponding to individual centers. Segment width is proportional to each center's patient count, and segment color indicates surgical volume: small (<20 patients, beige), medium (20–49, light orange), large (50–99, orange), and very large (≥100, dark orange).

We collected data on 2427 children and adolescents who underwent epilepsy surgery in 2023 from 61 epilepsy surgery programs in 29 countries across six continents (Figure 1, Table S1). Of these, 49 centers were pediatric‐only and 12 were mixed‐age centers with dedicated pediatric services. Patient distribution by continent was as follows: Asia (*n* = 960), Europe (*n* = 795), North America (*n* = 427), South America (*n* = 149), Oceania (*n* = 79), and Africa (*n* = 17). Annual surgical volume varied; 27 centers treated fewer than 20 patients (small volume), 22 treated 20–49 patients (medium), eight treated 50–99 patients (large), and four treated 100 or more (very large). The median number of patients per center was 24 (interquartile range = 12–45).

### 3.2 Rates and indications of invasive evaluations

**FIGURE 2:**
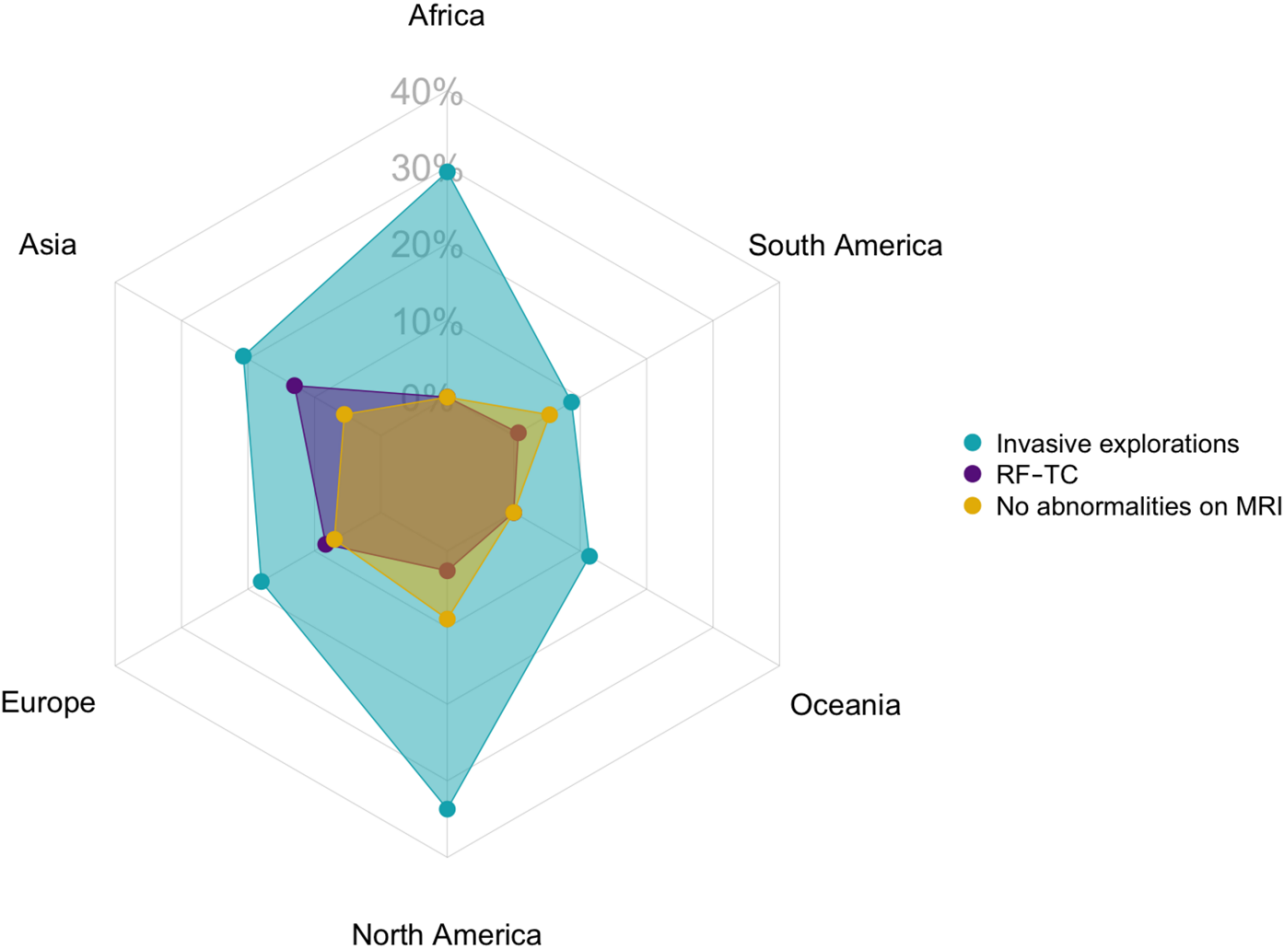
Invasive explorations, magnetic resonance imaging (MRI)‐negative patients, and stereoelectroencephalography (SEEG)‐guided radiofrequency‐thermocoagulation (RF‐TC). Invasive explorations were performed in approximately every fifth patient overall, with higher use in North America compared to Europe (*p* = .003). Overall, 6.4% of the cohort were MRI‐negative; these accounted for 32.3% of patients undergoing invasive exploration. SEEG‐guided RF‐TC was performed in 8.4% of the total cohort and in 40.9% of SEEG cases.

Invasive evaluations were performed in 21.1% of patients, with the highest rate in North America (33.7%) and the lowest in South America (8.7%; Figure 2, Table S2). Compared to Europe, invasive evaluations were more common in North America (GEE, *p* = .003) and less common in South America (*p* = .017).

Among those who underwent invasive evaluation, 32.3% had no MRI‐visible lesion (6.4% of all patients), with the highest rate in South America (61.5%, 8 of 13 patients) and no reported cases in Oceania (Figure 2, Table S2), without significant differences compared to Europe.

**FIGURE 3:**
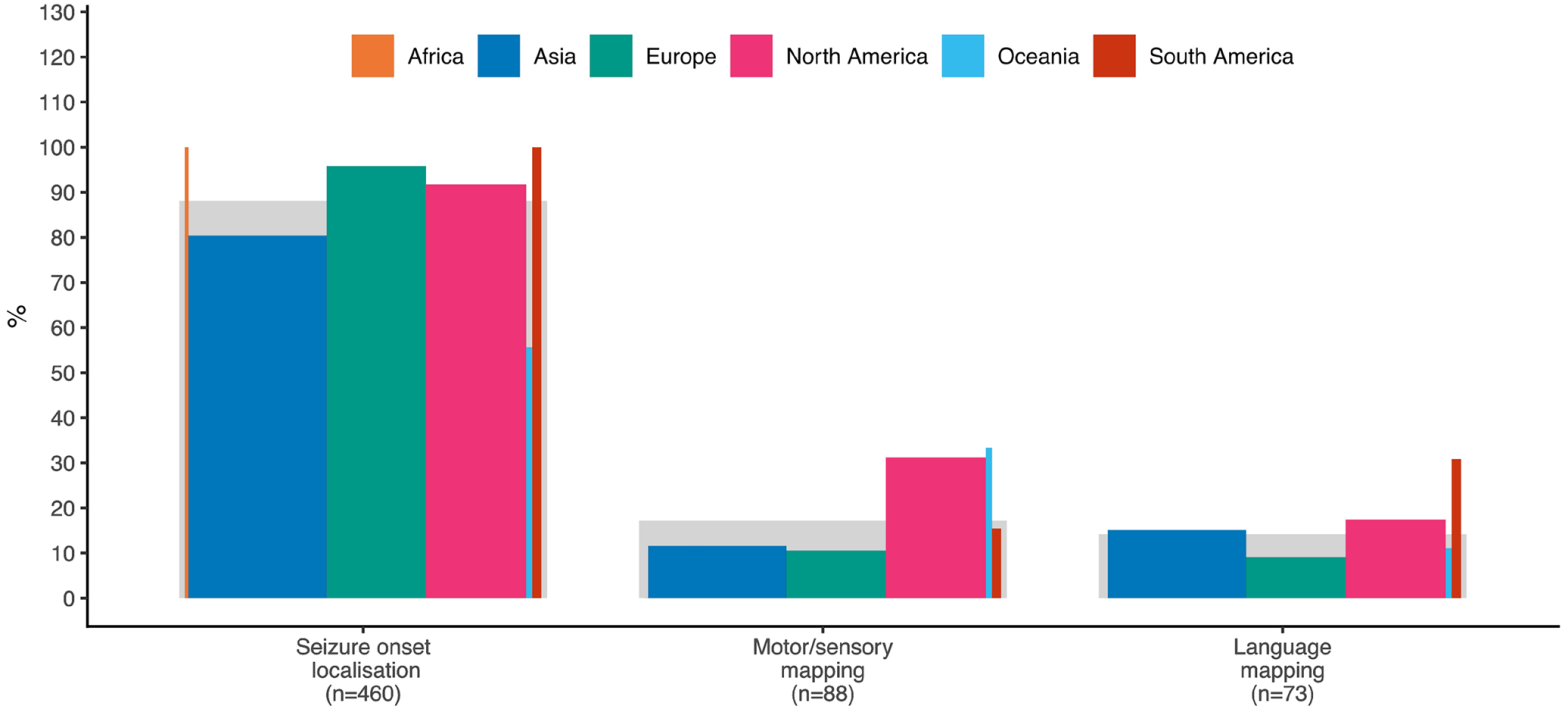
Indications for invasive presurgical evaluation vary across continents. Bar plots show the proportion of patients in each region who underwent invasive electroencephalography for (1) seizure onset localization, (2) motor/sensory mapping, and (3) language mapping. Colored bars represent continents: Africa (orange), Asia (dark blue), Europe (green), North America (pink), Oceania (light blue), and South America (brown). Bar width is proportional to the number of patients per region. Gray background bars indicate the overall global percentages. Globally, seizure onset localization was the most common indication (88.1%) for invasive exploration, whereas functional mapping was reported in 17.2% (motor/sensory) and 14.2% (language) of cases. Regional variation was observed, with particularly high rates of language mapping in South America and motor/sensory mapping in Oceania and North America.

The most common indication for invasive evaluation was localization of seizure onset in 88.1% of cases, highest in South America (100.0%, 13 of 13 patients) and lowest in Oceania (55.6%, five of nine patients; Figure 3, Table S2). Motor or sensory mapping was reported in 17.2% of cases overall, most often in Oceania (33.3%, three of nine patients) and least in Europe (10.5%). Language mapping was performed in 14.2% of cases, most frequently in South America (30.8%, four of 13 patients) and least often in Europe (9.1%).

### 3.3 Types of invasive exploration

**FIGURE 4:**
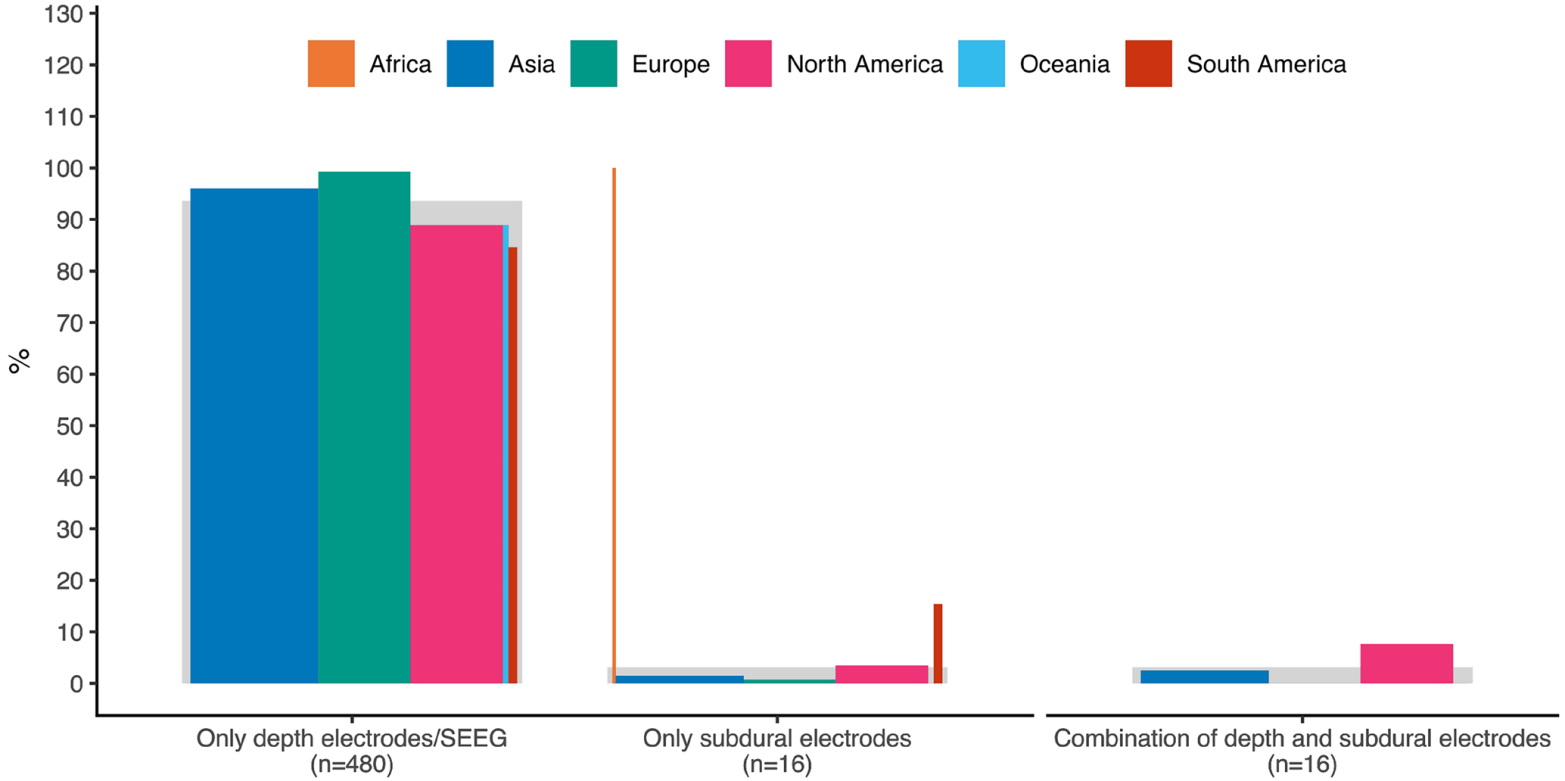
Modalities of invasive presurgical evaluation vary across continents. Bar plots show the distribution of invasive exploration modalities by region, categorized as the following: (1) only depth electrodes (stereoelectroencephalography [SEEG]), (2) only subdural electrodes (grids or strips), and (3) a combination of depth and subdural electrodes. Colored bars represent continents: Africa (orange), Asia (dark blue), Europe (green), North America (pink), Oceania (light blue), and South America (brown). Bar width is proportional to the number of patients per region. Gray background bars indicate global percentages. SEEG was the predominant modality globally, used in 93.6% of invasive cases (*n* = 507), whereas subdural‐only (3.1%, *n* = 16) and combined depth–subdural approaches (3.1%, *n* = 16) were rarely used.

SEEG was the most common modality, used in 19.8% of all patients (Figure 4, Table S3); rates were highest in North America (30.0%) and lowest in South America (7.4%). Compared to Europe, SEEG was more common in North America (GEE, *p* = .021). Overall, SEEG accounted for 93.6% of all invasive evaluations; only 3.1% were performed using subdural electrodes and 3.1% using combined depth and subdural electrodes.

Subdural electrodes were used in only .7% of all cases, most often in South America (1.3%), whereas no cases were reported in Oceania. Compared to Europe, both North America (*p* = .025) and South America (*p* = .030) reported higher utilization of subdural electrodes.

Combined depth and subdural electrodes were used in .7% of cases, with the highest rate in North America (2.6%), whereas no such cases were reported in Europe, Oceania, or South America.

SEEG‐guided radiofrequency thermocoagulation (RF‐TC) was performed in 8.4% of all patients and in 40.9% of SEEG cases, most commonly in Asia (63.8%), whereas no RF‐TC procedures were reported in Oceania (Figure 2, Table S3).

### 3.4 Outcomes of invasive evaluation

In 16.2% of invasive evaluations, patients did not proceed to resection, disconnection, or ablation. Rates ranged from 33.3% (three of nine cases) in Oceania and 31.5% (45 of 143 cases) in Europe to no such cases in South America (Figure 2, Table S3). Compared to Europe, North America had a significantly lower rate (GEE, *p* = .033).

## 4 DISCUSSION

This international survey provides the broadest overview to date of invasive presurgical evaluation in pediatric epilepsy surgery. It builds on the landmark 2004 survey,^10^ which included 543 patients from 20 programs across North America, Europe, and Oceania. In contrast, our dataset covers 2427 patients from 61 programs in 29 countries, including Asia, South America, and Africa, which were not represented in the earlier survey. This expanded coverage allows comparison of practices across participating centers and provides a broader international perspective on current invasive evaluation strategies, although these centers do not necessarily represent broader regional or continental practice, particularly in underrepresented regions such as Africa.

### 4.1 Utilization patterns and indications of invasive evaluation

Invasive evaluations were performed in 21.1% of patients in our survey, with the highest rate in North America (33.7%). This is slightly lower than the 27.0% reported in the 2004 ILAE survey,^10^ where invasive explorations were also more frequently used in North America than in Europe and Australia. Although advances in imaging and functional diagnostics may have reduced the need for invasive recordings at some centers, this effect may be offset by broader surgical indications, increasing case complexity, growing confidence in invasive electroencephalography (EEG), and expansion of programs performing invasive studies.^20^ A monocentric study from Germany similarly reported a decline in invasive evaluations over time, from 25.4% in 2000 to 18.9% in 2014,^21^ suggesting a regional trend in this direction.

MRI‐negative cases accounted for 32.3% of patients who underwent invasive evaluation in our cohort, with rates varying across regions, although some regional estimates were based on small numbers. In contrast, only 17.0% of surgical cases in the 2004 ILAE survey^10^ were MRI‐negative, with an additional 6.0% having subtle findings. This may reflect increased consideration of MRI‐negative or otherwise complex cases for surgery, supported by advances in imaging and SEEG planning. Although MRI‐negative epilepsy remains relatively uncommon among children undergoing surgery overall,^3^ it was clearly overrepresented among patients undergoing invasive evaluation.

Seizure onset localization was the main indication for invasive evaluation (88.1%), followed by motor/sensory (17.2%) and language mapping (14.2%). In the 2004 ILAE survey,^10^ seizure localization was also the main indication (92% among lesional cases), but functional mapping was more frequent: motor/sensory in 58.0% and language in 35.0% of cases. The lower rates of functional mapping in the current cohort may reflect increased reliance on noninvasive techniques and, in some centers, a preference for intraoperative functional monitoring (such as motor evoked potentials or awake procedures) rather than preoperative invasive mapping.

### 4.2 SEEG as the predominant invasive evaluation technique

SEEG was the predominant modality in our survey, used in 19.8% of all cases and in 93.6% of those undergoing invasive evaluation. Subdural and combined depth–subdural approaches were used in fewer than 1% of cases each, reflecting a near‐complete shift toward SEEG in pediatric epilepsy surgery across participating centers.^4^, ^9^ This contrasts with the 2004 ILAE survey,^10^ where subdural electrodes were the dominant approach. Originally developed in the 1950s by Talairach and Bancaud in Paris,^22^ SEEG remained limited to a few European centers until its global dissemination accelerated over the past 10–15 years, driven by advances in robotic implantation, vascular imaging, and EEG analysis.^23^ A 2022 US survey reported SEEG use at 92% of tertiary centers, with 76% favoring SEEG over subdural electrodes.^17^ The 2016 ILAE techniques survey^24^ similarly documented increasing global SEEG adoption and declining use of subdural electrodes. SEEG use varied by region in our survey, with the highest rates in North America and minimal use in South America, possibly reflecting disparities in training, infrastructure, and institutional practice.^25^, ^26^

As recently as 2016, ILAE recommendations emphasized a complementary role for both modalities.^20^ Subdural electrodes were considered preferable for extensive unilateral superficial epileptogenic zones requiring broad mapping,^27^ whereas SEEG was favored for deep, sulcal, or bilateral cases.^20^, ^28^ However, SEEG has since become the default invasive technique at many centers, supported by its practical advantages; it is less invasive, better tolerated, and associated with lower complication rates, while achieving comparable and in some series superior surgical outcomes.^18^, ^29^, ^30^, ^31^ In a multicenter study of 1468 patients, complications were significantly lower with SEEG (3.3%) than with subdural electrodes (9.6%).^30^ Whereas transfusions occurred in 13.0%–20.0% of pediatric subdural electrode cases, none was reported with SEEG.^32^, ^33^ Beyond technical aspects, SEEG and subdural electrodes also differ in clinical rationale. Subdural monitoring is typically performed to guide resection, whereas SEEG is often used to delineate epileptic networks and tailor individualized treatment strategies.^28^ Although fewer patients may proceed to resection after SEEG, seizure freedom rates have been reported to be higher than after subdural electrode studies.^30^

Although both techniques are feasible in children, SEEG presents technical challenges in those younger than 2–3 years due to insufficient skull thickness. A minimum bone thickness of 2 mm—typically reached by 18 months—is required for safe placement of anchor bolts.^28^, ^34^ Thin skulls increase the risk of bolt instability or skull fracture, requiring presurgical computed tomography with bone windows. However, SEEG has been safely performed in children as young as 16 months.^35^, ^36^ Even when minor postimplantation bolt shifts occurred, electrode trajectories remained accurate, with no complications reported. These findings suggest that skull thickness alone should not be considered an absolute contraindication. Robotic systems have further enhanced precision, enabling SEEG in selected infants.^36^

### 4.3 SEEG‐guided RF‐TC

SEEG‐guided RF‐TC was performed in 40.9% of SEEG invasive cases in our survey, reflecting growing interest in its use as both a diagnostic and therapeutic tool. First described in 2004,^37^ this technique uses the same depth electrodes implanted for SEEG to deliver targeted ablations within the epileptogenic zone. It is typically applied either to assess whether limited ablation of the seizure onset zone reduces seizures, thereby helping to predict the likely benefit of a larger resection, or as a palliative option for small, well‐defined, high‐risk targets, particularly in deep or eloquent regions.^38^, ^39^, ^40^, ^41^

Performing RF‐TC through existing SEEG electrodes eliminates the need for reimplantation and enables direct targeting based on ictal EEG patterns.^42^ Common targets include focal cortical dysplasia in central or insular regions^38^, ^43^ and periventricular nodular heterotopia, often with favorable outcomes. Some centers now apply RF‐TC routinely in selected patients undergoing SEEG.^39^, ^40^, ^41^ However, its utility in pediatric epilepsy surgery remains limited. A recent multicenter study found that only 21.3% of pediatric SEEG cases met criteria for RF‐TC.^43^ Younger children often present with broader or multifocal epileptogenic zones, where the small ablation volume of RF‐TC is insufficient to disrupt the network.^42^ In contrast, later onset epilepsy tends to involve more localized zones, making RF‐TC more feasible and potentially more effective.

RF‐TC can be considered in patients ineligible for resection, although long‐term seizure freedom is less likely.^42^ Selected pediatric series report short‐term seizure freedom of up to 71.0%, with transient neurological deficits in 19.4%, typically due to postablation edema resolving within weeks.^38^ In the largest pediatric cohort to date, involving mostly MRI‐positive focal cortical dysplasia cases, seizure freedom was 43.5% at 1 month and 26.7% at 1 year.^43^

Among RF‐TC responders, the positive predictive value for seizure freedom following subsequent resection was .83, supporting its role in surgical planning.^43^ Although laser interstitial thermal therapy (LITT) is gaining attention, RF‐TC remains widely used^38^ due to its bedside feasibility, low periprocedural complication rates (comparable to SEEG implantation alone), and lower cost. However, important limitations remain; RF‐TC produces small lesions and, unlike LITT, lacks real‐time imaging and volumetric thermal monitoring. The lesioning console limits energy delivery based on electrode‐tip thermometry (typically approximately 60°C near vessels and up to 75°C elsewhere), which limits precision and efficacy compared with LITT.

### 4.4 Outcomes of invasive evaluation

The decision to proceed with surgery after invasive evaluation depends on whether the epileptogenic zone can be satisfactorily localized and whether the expected benefit outweighs surgical risk.^30^ In our survey, 16.2% of patients who underwent invasive evaluation did not proceed to resection, disconnection or ablation, a lower rate than the 29.0% reported in the 2004 ILAE survey.^10^ Rates varied by region, with the highest rates in Oceania (33.3%) and Europe (28.5%), no cases in South America, and significantly lower rates in North America compared with Europe. The lower proportion compared with 2004^10^ may relate to advances in neuroimaging, invasive planning, and patient selection, which may have increased the likelihood that invasive evaluations lead to surgery. Given that seizure, functional, and other long‐term outcomes were not assessed, regional differences should be interpreted cautiously and may reflect variation in case complexity, selection strategies, thresholds for intervention, and health care context, including access to resources, reimbursement structures, and local risk tolerance. Long‐term data from a large European center have shown that roughly 40% of patients undergoing invasive evaluation did not proceed to surgery.^9^

Although a large multicenter study (*n* = 285) found no significant difference in surgical rates by type of invasive evaluation,^29^ other reports suggest that patients are less likely to undergo resection after SEEG than after subdural electrode studies.^30^, ^31^ This may relate partly to procedural logistics; for subdural electrodes, a second craniotomy is required for grid removal, potentially lowering the threshold for proceeding to resection, even in borderline or noncurative cases. In contrast, the lower morbidity of SEEG may lower the threshold for invasive evaluation, resulting in implantation of patients with a lower pretest probability of proceeding to resective surgery. Despite these concerns, our survey shows that the proportion of patients not proceeding to surgery after invasive evaluation has decreased markedly compared to the 2004 ILAE survey,^10^ despite SEEG now almost completely replacing subdural electrodes. This may reflect more effective preinvasive patient selection and improvements in invasive techniques, reducing implantations unlikely to lead to curative surgery. Nevertheless, the possibility that invasive evaluation will not lead to surgery should be addressed early in the decision‐making process and clearly communicated to families, given the burden of epilepsy and the nonnegligible risks associated with invasive EEG.^28^

### 4.5 Future directions

This study has limitations. Postsurgical seizure, functional, and developmental outcomes were not assessed, and group‐level data did not allow stratified analyses by age, etiology, MRI status, or invasive evaluation pathway. Future patient‐level studies should evaluate outcomes following invasive evaluation in these subgroups, including separately in MRI‐negative and MRI‐positive patients. Specific subgroup findings, particularly in underrepresented regions or small regional samples, should be interpreted cautiously, as they may be unstable and not generalizable.

## 5 CONCLUSIONS

This international survey provides the first broad overview of invasive presurgical evaluation practices in pediatric epilepsy surgery across participating centers worldwide. The findings confirm a near‐complete transition to SEEG, with minimal use of subdural or combined electrode approaches. SEEG is now widely used not only for seizure localization but also for functional mapping and RF‐TC, which is increasingly applied in selected high‐risk cases and as a tool to support surgical decision‐making. However, regional differences persist in access, utilization, and surgical pathways. The high proportion of MRI‐negative cases and the considerable proportion of patients not proceeding to resection underscore the complexity of contemporary surgical candidates and the need to further refine selection strategies. These findings also support continued efforts to improve access to advanced diagnostic tools, particularly in resource‐limited settings.

## AUTHOR CONTRIBUTIONS

Georgia Ramantani, Martha Feucht, Dorottya Cserpan, Dario Englot, Elaine Wirrell, J. Helen Cross, Manjari Tripathi, Howard Weiner, Mary Lou Smith, Lakshminarayanan Kannan, Lixin Cai, and Sarah Ferrand Sorbets contributed to the conception and design of the study. All authors contributed data for the article. Georgia Ramantani and Dorottya Cserpan analyzed the data, drafted the manuscript, and prepared the figures, with significant contributions from Martha Feucht, Lixin Cai, and Sarah Ferrand Sorbets. All authors reviewed, edited, and approved the final version of the manuscript.

## CONFLICT OF INTEREST STATEMENT

The authors declare no conflicts of interest. We confirm that we have read the Journal’s position on issues involved in ethical publication and affirm that this report is consistent with those guidelines.

## ETHICAL APPROVAL

The collection and analysis of patient data were approved by and performed according to the guidelines and regulations of the local ethics committee (KEK‐ZH Req‐2024‐00058).

## Supporting information


Table S1
Table S2
Table S3

## Data Availability

The data that support the findings of this study are available from the corresponding author upon reasonable request.
