# Supplementary material for: Pediatric epilepsy surgery: Global survey of invasive explorations
Source: Epilepsia. 2026 Jun 11;67(9):4470–82. doi: 10.1002/epi.70325 (PMC13592491; doi:10.1002/epi.70325)
Supplement: Supplementary file 1 [file EPI-67-4470-s001.docx]

**SUPPLEMENTARY TABLES**

| Institution | Responsible Physicians | Location |
| --- | --- | --- |
| ***Africa (n=17)*** |  |  |
| Mediclinic Constantiaberg | James Butler, Vicki Deli-Peri | Cape Town, South Africa |
| Red Cross War Memorial Children's Hospital | Jo Wilmshurst, Johannes M. Nico Enslin, Richard J. Burman | Cape Town, South Africa |
| ***Asia (n=960)*** |  |  |
| Epilepsy Center, Shenzhen Children's Hospital | Dezhi Cao | Shenzhen, China |
| Pediatric Epilepsy Center, Peking University First Hospital | Lixin Cai, Yuwu Jiang | Beijing, China |
| Krishna Institute of Medical Sciences | Sita Jayalakshmi, Manas Panigrahi | Secunderabad, India |
| Epilepsy Center, National Center of Neurology and Psychiatry | Masaki Iwasaki | Tokyo, Japan |
| All India Institute of Medical Sciences (AIIMS) | Manjari Tripathi, Poodipedi Sarat Chandra | Delhi, India |
| Deenanath Mangeshkar Hospital | Sandeep Bhagwan Patil | Pune, India |
| National Epilepsy Center, NHO Shizuoka Institute of Epilepsy and Neurological Disorders | Naotaka Usui | Shizuoka, Japan |
| Amrita Advanced Centre for Epilepsy (AACE), Amrita Institute of Medical Sciences, Amrita Vishwa Vidyapeetham | Siby Gopinath, Ashok Pillai | Kochi, Kerala, India |
| Osaka City General Hospital | Noritsugu Kunihiro, Takehiro Uda | Osaka, Japan |
| Gleneagles Hospital | Kannan Lakshminarayanan, Nigel Peter Symss | Chennai, Tamilnadu, India |
| P.D. Hinduja National Hospital & Medical Research Centre | Vrajesh Udani, Neelu Desai, Saheli Roy | Mumbai, India |
| Phramongkutklao Hospital | Piradee Suwanpakdee, Siraruj Sakoolnamarka | Bangkok, Thailand |
| Kyoto University Graduate School of Medicine | Takayuki Kikuchi | Kyoto, Japan |
| Osaka Metropolitan University Hospital | Takehiro Uda | Osaka, Japan |
| Avitis Institute of Medical Sciences | Dhanya T S, Kurupath Radhakrishnan | Palakkad, Kerala, India |
| Jichi Medical University Hospital | Kensuke Kawai, Naoto Kunii | Shimotsuke, Tochigi, Japan |
| National Epilepsy Center, National Hospital of Sri Lanka | Jithangi Wanigasinghe, Sanjaya Fernando, Sanjeewa Garusinghe | Colombo, Sri Lanka |
| ***Oceania (n=79)*** |  |  |
| The Royal Children's Hospital | Simon Harvey | Melbourne, Australia |
| The Children's Hospital at Westmead | Deepak Gill, Sachin Gupta | Sydney, Australia |
| Queensland Children's Hospital | Sameer Dal, Steve Malone, Kate Riney | Brisbane, Australia |
| ***Europe (n=795)*** |  |  |
| Great Ormond Street Hospital for Children | Helen Cross, Christin Eltze, Suresh Pujar, Martin Tisdall, Zubair Tahir, Friederike Moeller | London, UK |
| Royal Hospital for Children and Young People | Jay Shetty, Ailsa McLellan | Edinburgh, UK |
| Rothschild Foundation Hospital | Sarah Ferrand Sorbets, Georg Dorfmüller, Mathilde Chipaux Raffo | Paris, France |
| Bethel Epilepsy Center | Thomas Cloppenborg, Thilo Kalbhenn | Bielefeld, Germany |
| Hospital Sant Joan de Déu | Alexis Arzimanoglou, Javier Aparicio, Jana Domínguez, Alia Ramírez, Carlos Valera | Barcelona, Spain |
| The Children's Memorial Health Institute | Katarzyna Kotulska-Jóźwiak, Paweł Kowalczyk | Warsaw, Poland |
| University Medical Center Utrecht | Kees Braun | Utrecht, Netherlands |
| Medical University of Vienna | Martha Feucht, Karl Rössler | Vienna, Austria |
| Claudio Munari' Epilepsy Surgery Centre, ASST Niguarda Hospital | Laura Tassi, Veronica Pelliccia | Milan, Italy |
| Charles University and Motol and Homolka University Hospital and EpiReC Consortium | Martin Kudr, Pavel Krsek | Prague, Czech Republic |
| Bambino Gesù Children's Hospital | Nicola Specchio, Luca de Palma | Rome, Italy |
| Niño Jesús University Hospital | María Ángeles Pérez Jiménez, Marcelo Budke | Madrid, Spain |
| Tel Aviv Medical Center | Jonathan Roth, Shimrit Uliel-Sibony | Tel Aviv, Israel |
| Freiburg Epilepsy Center | Victoria San Antonio-Arce, Andreas Schulze-Bonhage | Freiburg, Germany |
| Center for Pediatric Neurology, Neurorehabilitation, and Epileptology, Schoen-Clinic | Tom Pieper, Till Hartlieb, Manfred Kudernatsch | Vogtareuth, Germany |
| Meyer Children's Hospital IRCCS, and University of Florence | Carmen Barba, Renzo Guerrini, Flavio Giordano | Florence, Italy |
| Clinical Hospital 'Alexandru Obregia' | Oana Tarta Arsene | Bucharest, Romania |
| Istituto Giannina Gaslini Children’s Hospital, IRCCS, University of Genoa | Lino Nobili, Stefano Francione, Giulia Nobile, Alessandro Consales | Genoa, Italy |
| University Children's Hospital Zurich | Georgia Ramantani, Niklaus Krayenbühl | Zürich, Switzerland |
| Queen Silvia Children’s Hospital, Sahlgrenska University Hospital | Tove Hallböök | Gothenburg, Sweden |
| La Timone Hospital, Aix-Marseille University | Fabrice Bartolomei, Didier Scavarda | Marseille, France |
| Department of Pediatric Neurology and Epilepsy Surgery Center, Marmara University | Dilşad Türkdoğan, Adnan Dağçinar | İstanbul, Turkey |
| University Hospital St. Ivan Rilski | Petia Dimova | Sofia, Bulgaria |
| Bethesda Children's Hospital | Lejla Vendegh, András Fogarasi | Budapest, Hungary |
| ***North America (n=427)*** |  |  |
| Texas Children’s Hospital | Howard Weiner, Kalman Katlowitz, Daniel Curry | Houston, TX, USA |
| The Hospital for Sick Children | Mary Lou Smith | Toronto, Canada |
| Nicklaus Children's Hospital | Matt Lallas | Miami, FL, USA |
| Epilepsy Center, Cleveland Clinic | Ahsan Moosa Naduvil Valappil, William Bingaman | Cleveland, OH, USA |
| Mayo Clinic | Elaine Wirrell | Rochester, MN, USA |
| David Geffen School of Medicine at UCLA | Aria Fallah, Cassia Maniquis | Los Angeles, CA, USA |
| British Columbia´s Children's Hospital | Mary Connolly, Mandeep Tamber | Vancouver, BA, Canada |
| CHU Sainte Justine, Université de Montréal | Aris Hadjinicolaou, Gianluca D'Onofrio, Alexander Weil | Montreal, Canada |
| ***South America (n=149)*** |  |  |
| Clinica Cukiert | Arthur Cukiert, Cristine Cukiert | Sao Paulo, Brazil |
| Hospital de Pediatría "Prof. Dr. Juan P Garrahan" | Roberto Caraballo | Buenos Aires, Argentina |
| Guillermo Grant Benavente Hospital | Carolina Munoz Castro | Concepcion, Chile |
| Hospital Carlos Van Buren, University of Valparaíso | Catalina Soto Órdenes | Valparaíso, Chile |
| Hospital Universitario San Ignacio | Juan Carlos Perez Poveda, Oscar Zorro Guio, Daniel Nariño González | Bogotá, Colombia |
| Fundación Hospital Pediátrico la Misericordia | Laura Guio Mahecha, Oscar Zorro Guio, Juan Carlos Perez Poveda, Daniel Nariño González | Bogotá, Colombia |
| Clínica Portoazul Auna | Juan Carlos Perez Poveda, Benedetti Isaac | Barranquilla, Colombia |

**Supplementary Table 1.** Pediatric epilepsy surgery centers and investigators participating in the 2023 ILAE survey.

| Region | Invasive explorations | No MRI-visible lesion | Reason for implantation | | |
| --- | --- | --- | --- | --- | --- |
|  |  |  | Seizure onset localization | Motor/sensory mapping | Language mapping |
| (Intercept) | 0.22 (0.13–0.36), p<0.001 | 0.42 (0.23–0.79), p=0.007 | 8.13 (3.22–20.52), p<0.001 | 0.16 (0.06–0.39), p<0.001 | 0.16 (0.07–0.34), p<0.001 |
| Asia | 1.13 (0.46–2.78), p=0.796 | 1.04 (0.35–3.1), p=0.949 | 0.5 (0.14–1.75), p=0.278 | 0.84 (0.19–3.65), p=0.820 | 0.53 (0.12–2.36), p=0.407 |
| North America | **2.32 (1.34–4.01), p=0.003** | 0.88 (0.33–2.36), p=0.795 | 1.14 (0.16–8.33), p=0.894 | 1.24 (0.42–3.61), p=0.698 | 2.55 (0.98–6.65), p=0.055 |
| Oceania | 0.59 (0.11–3.05), p=0.526 | not estimable | 0.22 (0.03–1.77), p=0.154 | 0.26 (0.04–1.88), p=0.182 | 0.86 (0.11–6.76), p=0.885 |
| South America | **0.4 (0.19–0.85), p=0.017** | 8.03 (0.5–129.04), p=0.141 | 0.97 (0.09–10.3), p=0.982 | 3.06 (0.68–13.79), p=0.145 | 1.05 (0.27–4.17), p=0.943 |

**Supplementary Table 2: Regional variation in invasive explorations and reasons for implantation.** Odds ratios (ORs), 95% confidence intervals, and p-values from generalized estimating equation (GEE) models assessing regional variation in invasive presurgical evaluation compared to Europe. The outcomes are proportions of patients undergoing invasive evaluation, those without MRI-visible lesions, and the main indications for implantation. Odds ratios greater than 1 indicate higher rates, and values below 1 indicate lower rates relative to Europe. Statistically significant differences (p < 0.05) are highlighted in bold. Africa was excluded from inferential analyses due to the small sample size (n = 17). The intercept reflects Europe’s baseline odds. When zero/sparse events caused separation, the contrast is **not estimable**; we report ‘not estimable’ and **omit p-values.**

| Region | SEEG | Subdural electrodes | RF-TC | Did not proceed to surgery |
| --- | --- | --- | --- | --- |
| (Intercept) | 0.22 (0.13–0.36), p<0.001 | not estimable | 0.67 (0.36–1.25), p=0.209 | 0.55 (0.22–1.38), p=0.200 |
| Asia | 1.13 (0.59–2.18), p=0.707 | 2.51 (0.19–32.73), p=0.483 | 2.28 (0.49–10.54), p=0.293 | 0.3 (0.06–1.54), p=0.149 |
| North America | **1.97 (1.11–3.51), p=0.021** | **9.41 (1.32–67.24), p=0.025** | 0.17 (0.03–1.1), p=0.063 | **0.19 (0.04–0.87), p=0.033** |
| Oceania | 0.52 (0.07–3.62), p=0.507 | not estimable | not estimable | 2.33 (0.24–22.36), p=0.463 |
| South America | **0.32 (0.18–0.59), p<0.001** | **11.14 (1.27–97.8), p=0.030** | **0.06 (0–0.7), p=0.025** | not estimable |

**Supplementary Table 3: Regional variation in the type of invasive implantation and its outcome.** Odds ratios (ORs), 95% confidence intervals, and p-values from generalized estimating equation (GEE) models assessing regional variation in specific invasive modalities and related outcomes, again compared to Europe. Included outcomes are the use of stereoelectroencephalography (SEEG), subdural electrodes, and SEEG-guided radiofrequency thermocoagulation (RF-TC), as well as the proportion of patients who underwent invasive evaluation but did not proceed to resection, disconnection, or ablation. No cases of combined depth and subdural electrode implantation were reported in Europe, prohibiting modality-specific comparisons. Odds ratios greater than 1 indicate higher rates, and values below 1 indicate lower rates relative to Europe. Statistically significant differences (p < 0.05) are highlighted in bold. Africa was excluded from inferential analyses due to the small sample size (n = 17). The intercept reflects Europe’s baseline odds. When zero/sparse events caused separation, the contrast is **not estimable**; we report ‘not estimable’ and **omit p-values.**
